# Mortuary and hospital-based HIV mortality surveillance among decedents in a low-resource setting: lessons from Western Kenya

**DOI:** 10.1186/s12889-022-12909-3

**Published:** 2022-03-29

**Authors:** Emmanuel Nyakeriga, Wanjiru Waruiru, Valarie Opollo, Anthony Waruru, Leonard Kingwara, Dickens Onyango, Muthoni Junghae, Sheru Muuo, Teresia Macharia, Catherine Ngugi, Mary Mwangome, Hammad Ali

**Affiliations:** 1 Global Programs for Research and Training, Surveillance Department, University of California, San Francisco, P.O. BOX 10203-00100, Nairobi, Kenya; 2 Kenya Medical Research Institute, HIV-Research Branch, Kisumu, Kenya; 3 Division of Global HIV & TB, Center for Global Health, Centers for Disease Control and Prevention, Nairobi, Kenya; 4 National AIDS and STIs Control Programme, Ministry of Health, Nairobi, Kenya; 5 Kisumu County Department of Health, Kisumu, Kenya; 6 Department of Global Health, Utrecht University, Utrecht, Netherlands; 7 Institute of Tropical Medicine, Antwerp, Belgium; 8 Division of Global HIV & TB, Center for Global Health, Centers for Disease Control and Prevention, Atlanta, GA USA

**Keywords:** HIV, Mortality, Decedents, Surveillance, Mortuary, Hospital-based, Lessons

## Abstract

**Background:**

Lack of dependable morbidity and mortality data complicates efforts to measure the demographic or population-level impact of the global HIV/AIDS epidemic. Mortuary-based mortality surveillance can address gaps in vital statistics in low-resource settings by improving accuracy of measuring HIV-associated mortality and indicators of access to treatment services among decedents. This paper describes the process and considerations taken in conducting mortuary and hospital-based HIV mortality surveillance among decedents in Kenya.

**Main text:**

We conducted HIV mortuary and hospital-based mortality surveillance at two of the largest mortuaries in Kisumu County, Kenya (April 16–July 12, 2019). Medical charts were reviewed for documentation of HIV status among eligible decedents. HIV testing was done on blood and oral fluid samples from decedents with undocumented HIV status and those whose medical records indicated HIV-negative test results > 3 months before death. A panel of experts established the cause of death according to the International Classification of Diseases, 10th Revision rules. Civil registry data for the year 2017 were abstracted and coded to corresponding ICD-10 codes.

Of the 1004 decedents admitted to the two mortuaries during the study period, 49 (4.9%) were unavailable because they had been transferred to other facilities or dispatched for burial before enrolment. Of the 955 available decedents, 104 (10.9%) were ineligible for the study. Blood samples were collected from 659 (77.4%) decedents, and 654 (99.2%) were tested for HIV. Of the 564 decedents eligible for the OraQuick® validation sub-study, 154 were eligible for oral sample collection, and 132 (85.7%) matched pre- and post-embalming oral samples were collected and tested. Of the 851 eligible decedents, 241 (28.3%) had evidence of HIV infection: 119 had a diagnosis of HIV infection recorded in their patient files, and 122 had serological evidence of HIV infection.

**Conclusion:**

This study shows that in low-resource settings, conducting hospital and mortuary-based surveillance is feasible and can be an alternative source of mortality data when civil registry data are inadequate.

**Supplementary Information:**

The online version contains supplementary material available at 10.1186/s12889-022-12909-3.

## Background

Accurate and timely data on cause of death (COD) is critical for guiding health programs and policies [1, 2]. COD data are pivotal in identifying the public health importance of various diseases, allocating resources for disease control, evaluating trends in mortality over time to assess the impact of national health programmes, and identifying disease determinants [3]. COD data are particularly important for measuring the population-level impact of the HIV epidemic and response [4, 5]. Studies have highlighted the need to strengthen mortality surveillance globally [6, 7], yet only approximately half of registered deaths have COD information [8].

Capturing data on all births and deaths, issuing birth and death certificates, and compiling and disseminating vital statistics, (including COD information [9]), is done using Civil registration and vital statistics(CRVS) systems which are also considered the gold standard for mortality surveillance [10, 11]. All low-income and two-thirds of middle-income countries have poor civil registration and health infrastructure [12, 13], or deaths are never registered [8]. In countries where death records are available, data quality is unreliable [14, 15] and COD is not systematically recorded [16–18], making it difficult to identify causal associations [19].

Survey methods can supplement vital registration in the absence of CRVS systems, when all deaths cannot be reported [20]. Prospectively, Mortality data can be collected through on-going surveillance or retrospectively by means of mortality surveys using verbal autopsy (VA). However, VA has limitations, especially for diagnosing illnesses with non-specific symptoms such as HIV which is generally asymptomatic for several years before development of AIDS-defining illnesses, malaria in adults, and diarrheal and acute respiratory infections in infants [15, 21]. Sensitive information around the COD maybe withheld from the VA interviewer in settings where stigma is high around a particular COD as is often the case for HIV [22].

HIV-related deaths have been estimated through standardized mathematical modelling, which relies on assumptions that are subject to uncertainty and hence limit the estimation of HIV-associated deaths [14, 23]. In high-income countries, high-coverage CRVS systems linked to HIV case-based surveillance are used to estimate HIV-related deaths [24]. Although national CRVS systems track COD, limited standardization of COD coding, unavailability of HIV status at time of COD certification, and the low estimated coverage of death reporting (~ 50%) limit the generalizability of these statistics [25]. Mortuary surveillance of cause-specific deaths can be used to understand the role of HIV/AIDS in mortality rates, compensate for inadequate mortality data, and supplement VA-generated data [26, 27]. Burial surveillance system coupled with VA is recommended where vital registration system are non-existent to track large-scale population-level interventions. Studies from Botswana [28], Côte d’Ivoire [14], Republic of the Congo [29], Ethiopia [30],South Africa [31],the United States [32], and Kenya [23] have used mortuary-based HIV surveillance (through complete, partial, or minimally invasive autopsies) as an alternative method for obtaining COD data. However, little data on similar application among infants and children are available, even though mortuary-based surveillance offers a valuable alternative method of obtaining information about COD for all age groups in the absence of an effective CRVS system.

In previous studies, enzyme-based assays have been used to detect HIV in post-mortem blood specimens [24, 29].

The main objective of this mortuary-based HIV surveillance study was to establish appropriate procedures for measuring HIV-associated mortality in Kisumu, Kenya (Kisumu County has a population of 1,144,777 [33] and has the second highest HIV prevalence (17.5%) in the country [34]) and to explore oral specimen collection as an alternative to blood samples among decedents. This paper presents a summary of the processes, considerations, field experiences, and lessons learned for implementing mortuary-based HIV surveillance activities. Detailed methodology and results for the mortuary-based HIV surveillance study are reported elsewhere [26, 35, 36].

## Main text

### Study context

In 2015, an HIV mortuary surveillance study was conducted in Nairobi in which blood was collected from decedents for HIV testing [37]. We conducted follow-up mortuary and hospital-based surveillance study in Kisumu (Western Kenya) to determine whether children could be included in HIV mortuary surveillance studies and whether the study could be replicated with similar success in other parts of Kenya with variations in the levels of urbanization, infrastructure, use of mortuaries, and HIV burden, we conducted a follow-up mortuary and hospital-based surveillance study in Kisumu (Western Kenya). This study was supported by the Ministry of Health through National AIDS and STI Control Programme (NASCOP), Kisumu County Department of Health, University of California, San Francisco (UCSF) and United States Centers for Disease Control and Prevention (CDC)-Kenya in collaboration with the Kenya Medical Research Institute (KEMRI) [26].

This cross-sectional study was conducted at mortuaries at Jaramogi Oginga Odinga Teaching and Referral Hospital (JOOTRH) and the Kisumu County Referral Hospital (KCRH). These mortuaries record three-quarters of the deaths reported in Kisumu County. Decedents admitted to these mortuaries were categorized as internal hospital deaths or as brought in dead. Decedents that required a post-mortem for legal reasons were designated as police cases and were also included in these two categories [26].

### Study timeline

**Fig. 1:**
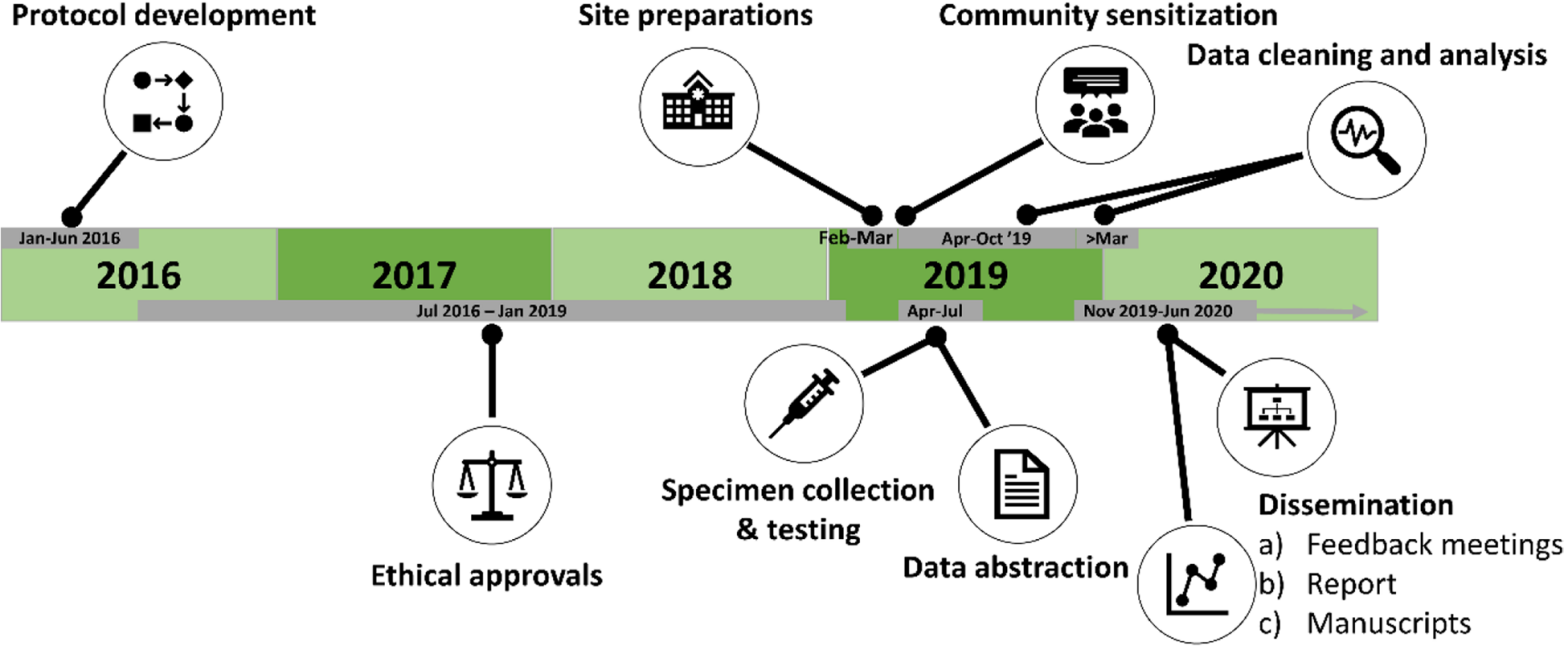
Timeline for a mortuary-based HIV surveillance study to establish appropriate procedures for measuring HIV-associated mortality and to explore oral specimen collection as an alternative to blood samples among decedents in Kisumu, Kenya (April 16–July 12, 2019)

It took 2.5 years to obtain all ethical approvals. Specimen collection was completed during the study period (April 16–July 12, 2019). Simultaneous to specimen collection, data abstraction and cleaning were carried out at the two hospitals and the civil registry. Community sensitization was carried out throughout the implementation through dialogue days in collaboration with the Kisumu County Community Health Units and presentations to the existing Community Advisory Boards. Figure 1 depicts the study timeline.

### Inclusion and exclusion criteria

Intact decedents of all ages admitted to JOOTRH and KCRH mortuaries during the study period (April 16–July 12, 2019) were included. However, for OraQuick® (OraSure Technologies, Bethlehem, PA USA) validation (exploring oral specimen collection as an alternative to blood samples among decedents), only eligible decedents aged ≥18 months admitted to JOOTRH mortuary were included. Decedents that had been dead for ≥48 h or whose blood could not be collected due to deterioration, burns, embalming, and stillbirths were excluded. Additionally, decedents having blood in the oral cavity as well as decedents < 18 months old were excluded for oral swab testing [26].

### Sample size

The sample size for the mortuary component of this study allowed estimation of HIV positivity among decedents aged ≥15 years with a margin of error of ±2% at alpha level of 95%, rounding up to 690 to account for a 20% anticipated loss of specimens. For oral sample testing for HIV, a purposive sample of specimens from 132 bodies (aged 18 months–adults) was used. Eligible decedents admitted to the two mortuaries were systematically enrolled in the study [26].

### Pre-study preparation

We conducted pre-implementation site visits. A standard checklist was used to assess and document various processes. To address gaps identified from these assessments, we purchased or repaired equipment and purchased supplies needed to conduct the study.

A total of six research assistants were contracted for 4 months; 34 nurses working at the two hospitals assisted with the retrieval of files; 15 mortuary attendants drew samples (blood and oral fluid); two pathologists provided supervisory support and seven laboratory technologists performed laboratory-based tests [26]. Medical officers and health records information officers were trained/retrained on COD certification and international classification of diseases and related health problems (ICD-10) coding rules [10]. A professional counsellor was contracted to provide counselling services to study staff.

### Field procedures

#### Hospitals

Research assistants at hospitals collected basic demographic information and HIV status of the decedent from medical files. If HIV-positive status or HIV-negative result of ≤3 months before death was documented in the medical records, neither blood nor oral fluid sample was drawn once cadaver was admitted to the morgue. If HIV status was undocumented or the records showed a HIV-negative result of > 3 months before death, the decedent was flagged for biological sample collection. To determine COD for hospital deaths, medical officers reviewed medical records of decedents enrolled in the study and abstracted history of illnesses, HIV status, and HIV treatment status. Medical officers determined the COD (immediate, antecedent, and underlying) for all decedents with a medical history. Health records information officers then assigned ICD-10 codes to the documented CODs, entered the CODs and respective ICD-10 codes into an Open Data Kit (ODK) electronic tool, and submitted the data to the study database [26].

#### Mortuaries

A non-clotted 6-mL blood specimen was collected using a percutaneous trans-thoracic 12-cm needle from each eligible decedent. Specimen collection was documented in a blood specimen collection register. Blood samples were triple packaged into a specimen cool box and within 4 h of collection were sent to the KEMRI HIV-R laboratory [26].

Pre- and post-embalming oral fluid samples were collected using the OraQuick® HIV test kit. The results were read after 20–40 min and interpreted according to the manufacturer’s instructions. For all cadavers admitted to the mortuaries, patient number and demographic and COD data were abstracted from the death notification forms (D1) or post-mortem reports, and data were entered into ODK and submitted daily to the study database [26].

#### Laboratory

Research assistants completed laboratory request forms at the mortuary to accompany blood samples to laboratory. A sample manifest form was used to track blood sample transport to the laboratory. Upon receipt of specimens at the laboratory, the specimen quality was verified against a pre-defined criterion. Dried blood spots (DBS) were prepared by spotting 1–2 drops (~ 70 μL) of blood drawn from EDTA tubes onto each of 5 spots of two filter papers per specimen (total of 10 spots). DBS cards were placed onto drying racks and left overnight to dry. Once dried, specimens were packed with desiccants and humidity indicator cards and stored at room temperature for up to 30 days before testing or at − 20 °C to − 30 °C for long-term storage awaiting antiretroviral metabolites testing. The remaining blood samples were centrifuged, and plasma was stored at 28 °C for up to 72 h before serological testing. All plasma that remained after serological testing was aliquoted into 2-mL cryovials, which were labelled and stored at − 80 °C for future testing [26].

Samples from infants aged < 18 months were tested for HIV via qualitative polymerase chain reaction. Samples from children aged 18 months–14 years, adolescents aged 15–18 years, and adults were tested for HIV antibodies, per the NASCOP HIV testing services guidelines [38]. All HIV-positive plasma samples were tested for VL (HIV-RNA copies/mL) via the Abbott m2000 system (Abbott Molecular, Des Plaines, IL USA). Where a plasma sample was not available, DBS were used for VL testing. VL results were reported quantitatively and categorized by viral suppression (< 1000/mL). Quality control for HIV serological testing was done daily by re-testing every seventh HIV-negative specimen using HIV rapid test kits used for the study. The laboratory also participated in a proficiency testing program for both HIV rapid and VL tests. Laboratory results were transcribed on a paper laboratory reporting form then entered into the electronic data collection tools on ODK and submitted to the study database [26].

#### Kisumu civil registry

At the Kisumu East Department of Civil Registration, COD was abstracted using ODK (Additional file 1) and submitted to the study database from available D1(death registration form completed by qualified medical personnel who certify the COD from all hospital deaths or post-mortem records; Additional file 2) and D2 forms (death registration form completed by the assistant chief who reports the COD for all deaths that occur in the community; Additional file 3) for all patients who died at the two hospitals. To annualize the COD for Kisumu East and to account for both data completeness and existing data entry backlogs, we selected records from 2017 for abstraction [26].

### Data management and analysis

#### Tools

**Fig. 2:**
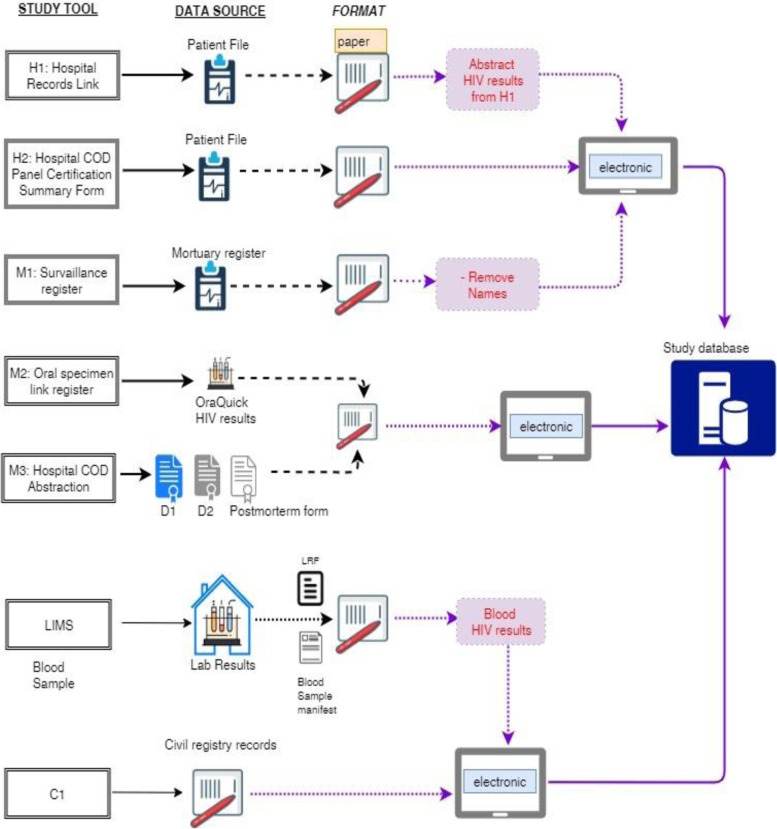
Data tools and flow for a mortuary-based HIV surveillance study to establish appropriate procedures for measuring HIV-associated mortality and to explore oral specimen collection as an alternative to blood samples among decedents in Kisumu, Kenya (April 16–July 12, 2019)

A hospital records link sheet (Additional file 4) was developed to abstract data from hospital deaths. A paper register was created to capture details of all deaths documented in the mortuaries during the study period and also served as the blood collection register (Additional file 5). An oral fluid collection register (Additional file 6) was used to record details relating to oral HIV testing. A tablet-based ODK form was developed to enter data abstracted from the two registers. A COD data abstraction form (Additional file 7) was used to enter COD data abstracted from the D1 forms (Additional file 2). For each blood sample, a laboratory request form (Additional file 8) was used, and a manifest sample form (Additional file 9) was used to track sample transport [26]. For COD certification, a COD panellist summary form (Additional file 10) was developed to record immediate, antecedent, and underlying COD. Paper-based study tools were securely kept in lockable cabinets and were accessible only to study staff during the study period [26]. Figure 2 outlines the different data sources and tools and the flow of data to the study database.

#### Data management and quality

All data collected on paper-based registers were abstracted into electronic format and submitted to the study database using ODK. Logic checks were built into electronic tools, double-data entry was done for selected variables, and data quality was audited. Data quality assessments (DQA) were done periodically on 10% of the data from the mortuary registers, COD abstraction forms, laboratory results, and abstracted civil registry data. Additionally, a random selection (10%) of entries made at the civil registrar’s office were re-abstracted and compared against initial entries [26].

#### Data analysis

Data were analysed in STATA (Stata Corporation, College Station, TX USA), version 14. Civil registry data were summarized to calculate the distribution of deaths by demographic and clinical variables; deaths that had a missing or invalid cause were excluded, including deaths where only mode of death was provided (e.g., cardiopulmonary failure or old age). COD data were analysed using *analysing mortality levels and causes-of-death* (ANACoD) tool, version 2.0 [39]. The tool also classified the underlying COD using the Global Burden of Diseases (GBD) categorisation and provided a comparison of findings with those from other countries [39]. We used the GBD approach of grouping deaths by broad causal categories to classify COD: Group I included communicable, perinatal, maternal, and nutritional diseases; Group II included non-communicable diseases; and Group III included injuries. Group I was further categorised into HIV-related and non-HIV-related causes [40].

### Ethical considerations

The study was approved by KEMRI’s Science and Ethical Review Unit (KEMRI/RES/7/3/1), JOOTRH ethics committee (ERC.IB/VOL.1/615), and the UCSF Committee for Human Research (230355).The study also was reviewed in accordance with CDC human research protection procedures and was determined to be research involving data or specimens from deceased persons [26].

## Results

### Enrolment

**Fig. 3:**
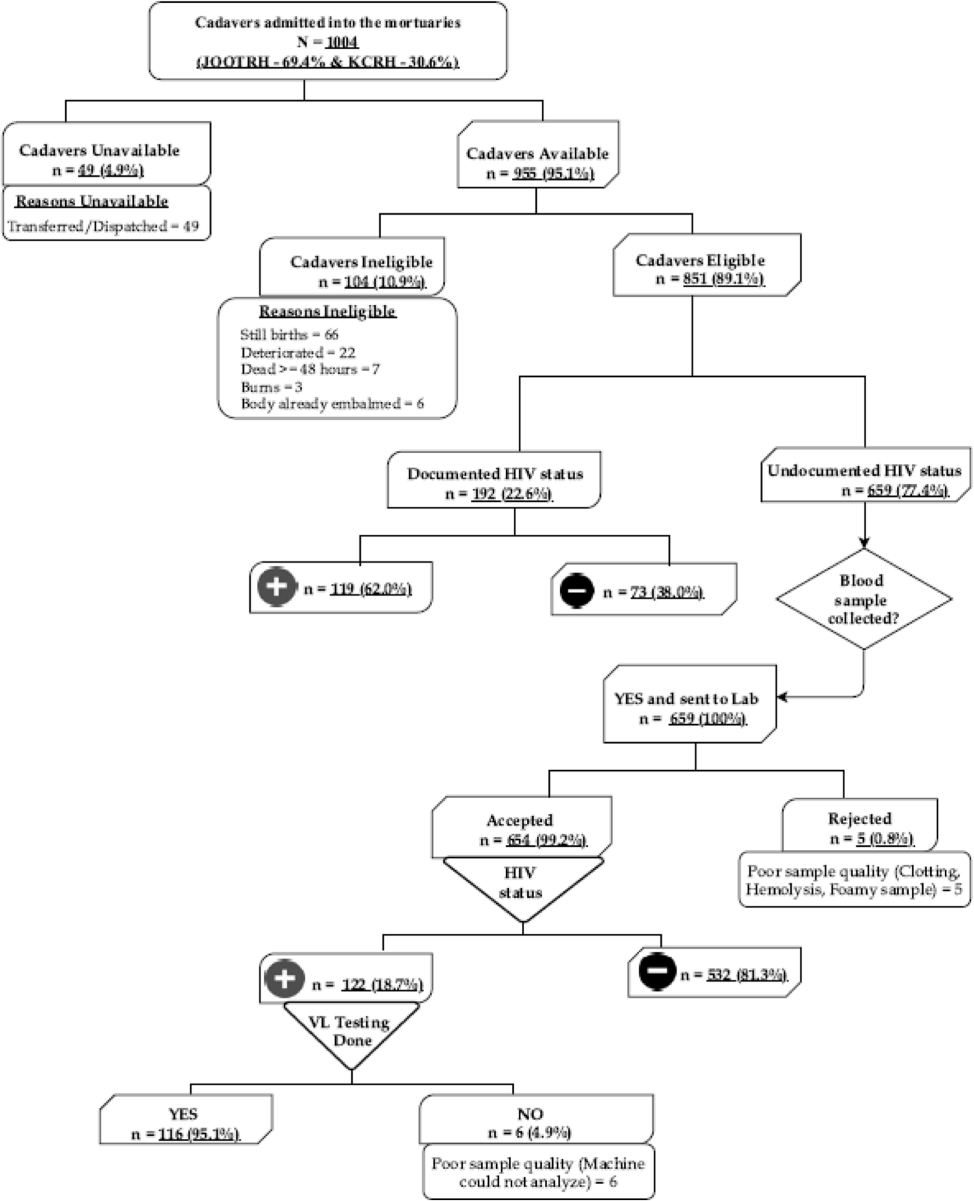
Enrolment flow chart for a mortuary-based HIV surveillance study to establish appropriate procedures for measuring HIV-associated mortality and to explore oral specimen collection as an alternative to blood samples among decedents in Kisumu, Kenya (April 16–July 12, 2019)

Of the 1004 decedents admitted to the two mortuaries during the study period, 697 (69.4%) were from JOOTRH, and 307 (30.6%) were from KCRH. Among the admitted decedents, 49 (4.9%) were not available because they were subsequently transferred to other facilities or dispatched for burial before enrolment. Of the 955 available decedents, 104 (10.9%) were not eligible for the study. The most common reason for ineligibility was stillbirth (66 [63.5%]; Fig. 3).

### General characteristics of eligible decedents

Most 555 (65.2%) of the eligible decedents were internal hospital deaths (JOOTRH and KCRH);of those brought in dead, 42 (14.2%) were police cases. Of the eligible decedents, 439 (51.6%) were men; of those aged< 15 years (161 [18.9%]), 94 (58.4%) were aged < 18 months. Among decedents aged ≥75 years, there were twice as many women as men (Table 1).

**Table 1:**
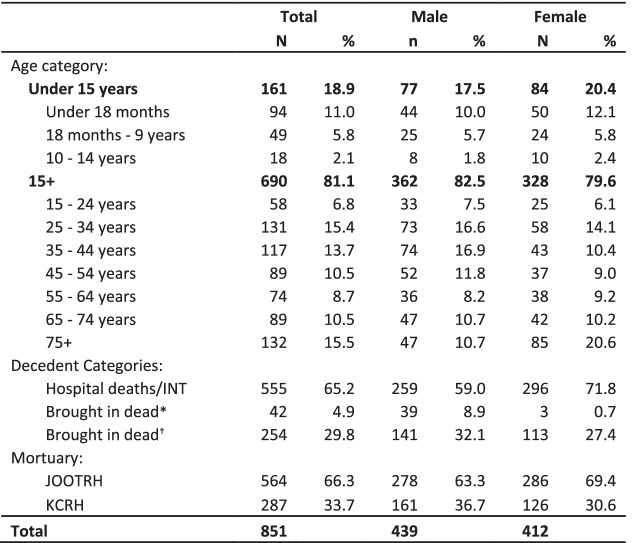
General Characteristics of the eligible decedents in a mortuary-based HIV surveillance study to establish appropriate procedures for measuring HIV-associated mortality and to explore oral specimen collection as an alternative to blood samples among decedents in Kisumu, Kenya (April 16–July 12, 2019)

*These include police cases e.g., homicides

^†^These include community-based deaths, transfers from other mortuaries but exclude police cases

### OraQuick®validation sub-study at JOOTRH

**Fig. 4:**
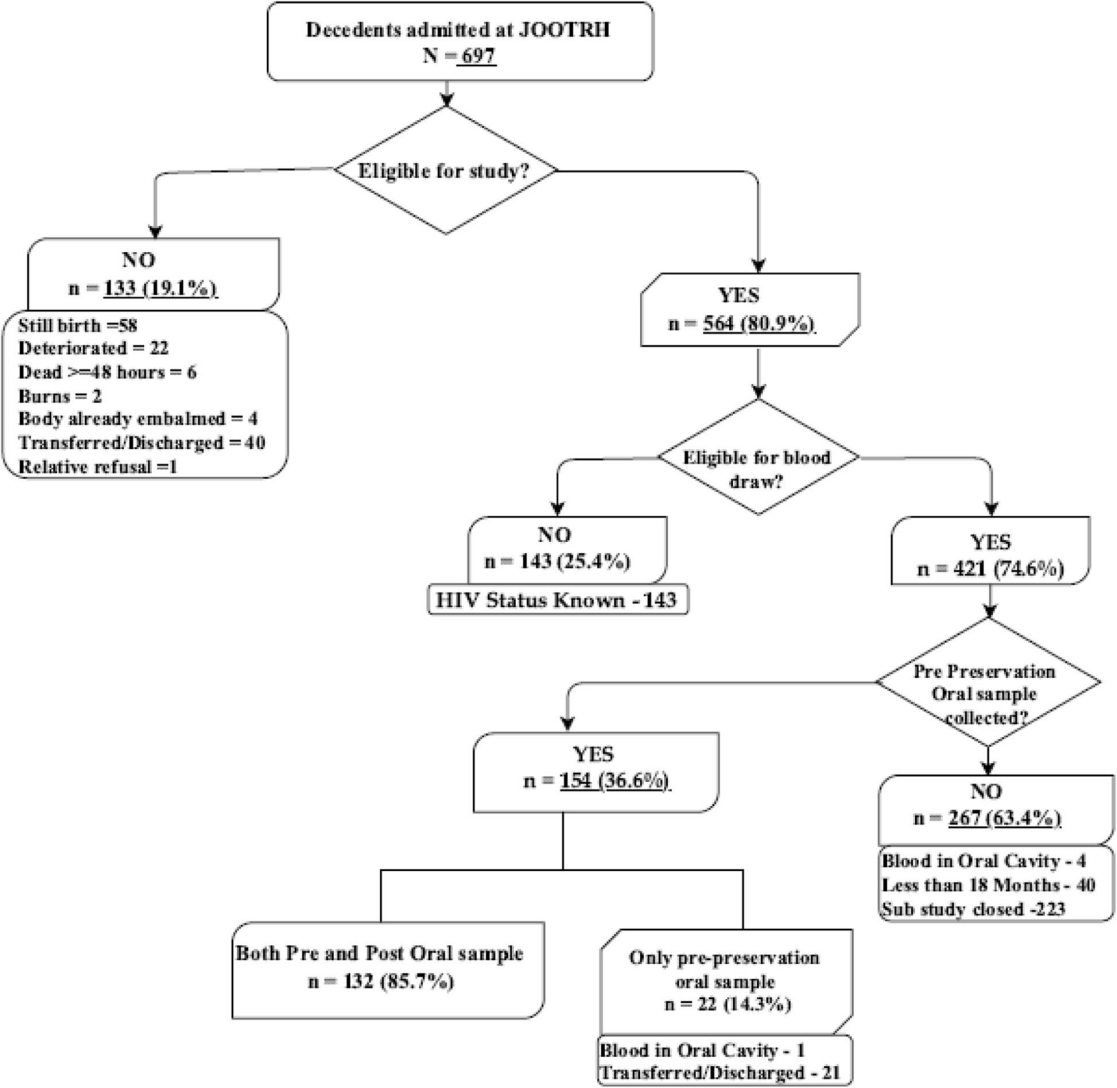
Flow chart enrolment to OraQuick®sub-study in a mortuary-based HIV surveillance study to establish appropriate procedures for measuring HIV-associated mortality and to explore oral specimen collection as an alternative to blood samples among decedents in Kisumu, Kenya (April 16–July 12, 2019)

Of the 697 decedents admitted into JOOTRH, 564 (80.9%) were eligible for the OraQuick®validation sub-study; of these, 421 (74.6%) were eligible for blood and oral fluid sample collection (Fig. 4). Samples from 267 (63.4%) eligible decedents were not collected because blood was present in the oral cavity, the decedent was aged < 18 months, or the sub-study had closed. Of the remaining 154 decedents, 132 had matched pre-embalming and post-embalming samples, and 22 decedents only had pre-embalming samples collected as they had either been dispatched or had blood in their oral cavities (Fig. 4).Detailed results for the OraQuick® Validation Sub-Study at JOOTRH are reported elsewhere [26, 36].

### Documentation of HIV status

Of the 851 eligible decedents, 192 (22.6%) had HIV status documented in the patient file. Of these, 119 (62.0%) were HIV positive. Blood samples were drawn from 659 decedents with undocumented HIV status for analysis in the laboratory out of which and 122 had serological evidence of HIV infection (Fig. 3). Out of the 555 files for eligible decedents who had died at the two participating hospitals (hospital deaths), 456 (82.2%) files were retrieved for certification of COD and the expert panel captured the HIV status of cadavers in 234 (51.3%) files [26].

### Blood and oral samples quality

Although samples were drawn from decedents who had died within 48 h, only 5 (0.8%) blood samples were of poor quality (clotted, haemolysed, or foamy) and were thus excluded while 654 (99.2%) were tested for HIV. For oral post-embalming samples, 4 (0.03%) samples were not collected from decedents because there was blood in the oral cavity after embalming.

## Discussion

Our study shows that with proper planning, sound coordination, stakeholder engagement, and study staff training, mortuary surveillance can help provide important COD data. Even in low-resource settings, mortuary surveillance is feasible to conduct a hospital and mortuary-based surveillance for decedents of any age. We achieved the targeted sample size within a relatively short period (3 months).With proper staff training and defined eligibility criteria, minimum sample rejections occur in samples drawn from decedents. Collecting samples for HIV testing as soon as the decedents are admitted to mortuaries is important because some decedents will often be transferred to other mortuaries or be discharged for burial.

Studies conducted among decedents navigate complex ethical questions [41] that could delay protocol approvals by respective ethics committees. It took longer than anticipated for this study to address concerns that were raised by respective ethics committees. Studies on decedents that are culturally sound and implemented as guided by country legislations, policies, and stakeholder engagements can help ensure that data are collected ethically and that human subjects are protected. Understanding communities’ concerns and reservations for such research can facilitate mortuary surveillance. Evidence-based communication tools are vital to educating the public regarding the importance of research and routine data collection involving people who have died [14]. Accounting for requirements and lead times for ethical approvals can facilitate mortuary surveillance implementation. Engaging stakeholders from conceptualization to dissemination of findings also facilitates mortuary surveillance. Support from both the national Ministry of Health and County Department of Health was crucial for our study.

Visits to the mortuary before implementation are key in pre-testing surveillance tools and identifying gaps that could jeopardize implementation of HIV mortuary surveillance activities. For example, in low-resource settings, mortuaries face infrastructural challenges, such as dysfunctional sinks, body trolleys, and waste biosafety supplies; these supplies and equipment could be covered by study budgets. Weak infection prevention and control measures in mortuary settings, including inadequate or inconsistent use of personal protective equipment and poor waste management practices, could be mitigated in HIV mortuary surveillance studies that may also strive to build capacity among mortuary staff on biosafety practices and emphasizing the importance of adhering to existing safety practices thus help mitigate potential risks.

Our study also was successful because we had detailed analysis and output plans, and we analysed COD data using freely available tools such as ANACoD that gave us the flexibility to re-analyse data as needed. Technical expertise, experience in diagnostic settings, and willingness to work in the mortuary setting are key selection criteria for research assistants. Providing guidance and counselling sessions to our study team helped staff acclimate to the mortuary setting. Future studies could consider training additional staff members to serve as replacements if needed during study implementation. A panel of clinicians, such as the panel used in the Child Health and Mortality Prevention Surveillance (CHAMPS) study [42], could help ensure accuracy of COD determination. The current supervision routinely offered to mortuary attendants by mortuary superintendents and pathologists could be utilized to support sample collection for similar mortuary surveillance studies. HIV testing service providers could be stationed at the mortuaries to test collected oral fluid specimens. In our study, the proximity of the two mortuaries and the laboratory facilitated overall study coordination.

A substantial percentage of patient files could not be retrieved, and for those that were retrieved, some were missing indicators of interest (i.e., HIV status, ART status, and VL). In addition, patients’ details (age and date of birth) captured in source documents are often inconsistent. Data management for this study was relatively complex due to the various data sources, formats, and coordination of timing when data were needed for critical decision making (for example, ascertaining HIV status of a decedent from hospital records to determine if a sample was needed before embalming). Abstraction of the COD from the civil registry sometimes took longer as paper-based records were either in use by registry officers or could not be easily located. Data completeness and data entry backlogs were also a hindrance to annualizing the most current COD for Kisumu East; therefore, the most current civil registry data (2018/19) could not be used. These challenges are likely to be present in many developing countries where CRVS systems are neither well established nor available electronically.

Without electronic medical records, more time is needed to retrieve specific physical medical files for decedents. Confirming HIV status documentation as soon as the bodies are admitted to the mortuaries could help determine whether a sample should be taken from the decedent. Optimizing this process could help ensure all eligible decedents are enrolled in mortuary surveillance.

Specific patient data may not be available in inpatient files because medical records could be stored at comprehensive care clinics (which provide free care for patients with retroviral disease); thus, some records may be available within hospital documents but are absent in-patient files. Delays in completing and submitting D1s coupled within sufficient or inconclusive data, increases use of undefined conditions (e.g., cardiovascular disease, respiratory disease, or cardiac arrest) as COD and minimize the public health use of data.

Decedents are often transferred to different mortuaries based on preferences of the decedents or their relatives. Alerting study teams when decedents are moved and coordinating enrolment processes could help avoid duplications and associated costs for duplicated tests. Considering these challenges can help determine how many research assistants are needed to support implementation of mortuary surveillance studies. Our study has several limitations that could be addressed in future surveillance activities. First, our study primarily used public mortuaries, which excluded private mortuaries and decedents who may be from a higher socioeconomic status. Secondly, reported number of stillbirths may not be representative of the true incidence of stillbirth in the study population and is not necessarily a reflection of quality of antenatal care at these facilities. Thirdly, medical history was only available for hospital-based deaths, and research assistants were not able to locate 18% of the inpatient files.

A total estimated cost of $100,609 is needed to conduct a similar mortuary and hospital-Based HIV mortality surveillance activity. An approximate cost of $48,474 was for various laboratory tests (genotyping, viral load, polymerase chain reaction, OraQuick® and rapid HIV tests) as well as equipment and consumables (cadaver movement trolleys, improving the plumbing systems, personal protective equipment, specimen collection and storage materials). Additional estimated $29,250 was spent on personnel related costs as the study required a variety of technical skills. This cost included personnel salaries, stipends and research allowances. A further estimated $22,885 was spent on various administrative costs (pre-survey, coordination and post-survey activities). Respective pre-survey activities costed $5870 and these included ethical submission fees, per diems, printing, communication, conference packages and transport (air and road) related costs. The study also incurred additional $10,515 for various coordination related tasks (transport, communication, logistics for shipping samples, sample storage, laptops, tablets, per diems and allowances). Post survey activities costing a total of $6500 were carried out at the end of the surveillance activity and these included holding a report writing workshop ($2800), printed the final report ($1200) as well as it launching and dissemination ($2500). Structured discussions with respective ministries of health on how such activities could be embedded and supported as part of routine surveillance activities could enhance sustainability for future implementations [26].

The results from this study depict an expansion of surveillance to a higher HIV burden region in Kenya adding the knowledge of prevalence patterns and analysis of impact of widespread ART coverage. This study illustrates that a mortuary and hospital-based surveillance system can indeed allow for an assessment of how HIV status is documented in medical records, assess and standardize consenting procedures and provide guidance for authorization for HIV testing of cadavers. The parallel specimen testing using OraQuick® as a rapid test provide additional evidence on reliability of a non-invasive alternative that may make it easier to implement mortuary and hospital-based HIV-associated mortality surveillance in sentinel sites. The findings from this study will be pivotal for writing a guideline for mortuary and hospital-based surveillance of HIV-associated mortality in Kenya. Additionally, these results will be used by the Kenya Ministry of Health to further support the roll out mortuary and hospital-based HIV-associated mortality surveillance in selected sentinel surveillance sites in Kenya as part of broader HIV surveillance.

## Conclusion

Mortuary and hospital-based surveillance is feasible in low-resource settings. Innovative HIV cost reducing mortality surveillance approaches, such as using oral fluid swab specimens for HIV testing obtained from decedents, can be used to provide much-needed public health insight about disease distribution and impact, especially in low-resource settings, and can help assess a country’s progress toward HIV epidemic control. Community sensitization and approval, buy-in by authorities and ethical, cultural, and logistical aspects are important considerations in planning mortality surveillance studies in similar settings. Electronic tools such as ANACoD can be used for analysis of COD data from HIV mortality surveillance and provide useful comparisons of COD statistics across countries.

## Supplementary Information

**Additional file 1.** County Civil Registrar Data Collection Form. ODK electronic tool.

**Additional file 2.** Death Notification Form (D1). Death registration form filled by qualified medical personnel who certify the cause of death from all hospital deaths or post-mortem records).

**Additional file 3.** Death Notification Form (D2). Death registration form filled by the assistant chief who reports the cause of death for all deaths that occur in the community).

**Additional file 4.** HospitalRecordsLink Sheet. Used to abstract data from hospital deaths, which were subsequently entered into an Open Data Kit (ODK) form.

**Additional file 5.** Mortality Surveillance Register. A paper register created to capture details of all deaths documented in the mortuaries during the study period and doubled up as the blood collection register.

**Additional file 6.** Mortality Surveillance Oral Sample Register. Recorded details relating to oral HIV testing for all eligible decedents at JOOTRH mortuary.

**Additional file 7.** Cause of Death (COD) data Abstraction Form. Used to enter COD data abstracted from the D1 forms.

**Additional file 8.** Mortality Study Laboratory Requisition Form. Used for requesting specific laboratory tests to be conducted on the sample at the KEMRI HIV-R laboratory.

**Additional file 9.** Sample Manifest for Blood Specimen. Used to track sample transport to the laboratory.

**Additional file 10.** COD panelist summary form. Used to record immediate, antecedent and underlying causes of death.

## Data Availability

The data used for this study are presented in the main manuscript.

## Abbreviations

ANACoD: Analysing mortality levels and Causes-of-Death
CDC: Centers for Disease Control and Prevention
COD: Cause of Death
CRVS: Civil Registration and Vital Statistics
D1: Death notification form for reporting deaths that occur within a hospital
D2: Death notification form for reporting deaths that occur in the community
DBS: Dried Blood Spots
EDTA: Ethylenediaminetetraacetic Acid
HIV/AIDS: Human Immunodeficiency Virus infection/Acquired Immunodeficiency Syndrome
HIV-R: Human Immunodeficiency Virus Research
ICD-10: International Classification of Diseases, 10th Revision
JOOTRH: Jaramogi Oginga Odinga Teaching and Referral Hospital
KCRH: Kisumu County Referral Hospital
KEMRI: Kenya Medical Research Institute
NASCOP: National AIDS and STI Programme
ODK: Open Data Kit
VL: Viral Load
