# Supplementary material for: Mortuary and hospital-based HIV mortality surveillance among decedents in a low-resource setting: lessons from Western Kenya
Source: BMC Public Health. 2022 Mar 29;22:609. doi: 10.1186/s12889-022-12909-3 (PMC8962591; doi:10.1186/s12889-022-12909-3)
Supplement: Supplementary file 2 — Additional file 2. Death Notification Form (D1). Death registration form filled by qualified medical personnel who certify the cause of death from all hospital deaths or post-mortem records). [file 12889_2022_12909_MOESM2_ESM.pdf]

Death Notification Form (D1)   v 16.08.18

FORM D1

REPUBLIC OF KENYA

THE BIRTHS AND DEATHS REGISTRATION ACT
(*Cap.* 149)

PERMIT FOR BURIAL

Serial No. DA   IP Number ....

1. NAME OF DECEASED ....
First Name   Middle Name   Father's or husband's name

2. IDENTIFICATION /PASSPORT NUMBER........................

4. SEX: Male ☐ Female ☐ 5. AGE........................ 6. DATE OF DEATH ....
Year s Month s Days   Day Month Year

9. USUAL RESIDENCE ....
Sub-location or estate and town   District

After making due inquiry as to cause of the death of the above named deceased person. I hereby authorize the interment of the body.

18. DATE:........................ 19. REGISTRATION ASSISTANT FOR: 20. SIGNATURE
Day Month Year

....

PERMIT ISSUED TO (NAME): .... ID No. .... SIGNATURE ....

FORM D1

REGISTER OF DEATH

(for use in health institutions and by Medical Practitioners)

Serial No. DA   IP Number ....

1. NAME OF DECEASED ....
First Name   Middle Name   Father's or husband's name

2. IDENTIFICATION /PASSPORT No........................ 3. NATIONALITY........................

4. SEX: Male ☐ Female ☐ 5. AGE........................ 6. DATE OF DEATH........................
Years months days   Day Month Year

7. MARITAL STATUS: (a) Married ☐ (b) Divorced (c)Single ☐ (d) Widowed ☐

8. PLACE OF DEATH:......................../........................
Health Institution/Sub-location or estate and town.   District

9. USUAL RESIDENCE......................../........................
Sub-location or estate and town   District

10. LEVEL OF EDUCATION ....11. OCCUPATION ....

MEDICAL CERTIFICATION

12. CAUSE OF DEATH (PRINT IN BLOCK LETTERS, DO NOT ABBREVIATE) ....

IMMEDIATE CAUSE: disease or condition directly leading to death (a)........................

Due to

ANTECEDENT CAUSES: Morbid conditions, if any, which gave rise to immediate cause (a)

(b)........................

Due to stating the underlying condition last

(c)........................

OTHER SIGNIFICANT CONDITIONS: Contributing to death but not related to (a) ....

13. CERTIFICATE: I certify that:

Tick as Appropriate

(a) I attended the deceased before death or
(b) I examined the body after death; or
(c) I conducted a post-mortem examination of the body, and that the above information is correct to the best of my knowledge.

14. NAME........................15. TITLE........................

16. DATE........................17. SIGNATURE........................

REGISTRATION ASSISTANT

18. DATE........................19. REGISTRATION ASSISTANT FOR: 20. SIGNATURE........................
Day Month Year   (Name of health institution)

....

REGISTRAR

21. DISTRICT........................22. REGISTRATION No........................

23. DATE........................24. NAME........................ 25. SIGNATURE........................
