# Supplementary material for: Mortuary and hospital-based HIV mortality surveillance among decedents in a low-resource setting: lessons from Western Kenya
Source: BMC Public Health. 2022 Mar 29;22:609. doi: 10.1186/s12889-022-12909-3 (PMC8962591; doi:10.1186/s12889-022-12909-3)
Supplement: Supplementary file 3 — Additional file 3. Death Notification Form (D2). Death registration form filled by the assistant chief who reports the cause of death for all deaths that occur in the community). [file 12889_2022_12909_MOESM3_ESM.pdf]

Death Notification Form (D2) v 16.08.1

REPUBLIC OF KENYA
THE BIRTHS AND DEATHS REGISTRATION ACT
(*Cap.* 149)
REGISTER OF DEATH **FORM D2**
(*For use by Registration Assistants for home deaths*)

Serial No. ____________________

1. NAME OF DECEASED ____________________
First Name ______ Middle Name ______ *Father's or husband's name ______

2. IDENTIFICATION /PASSPORT NO ____________ 3. NATIONALITY ____________
(*ID to be surrendered*)

4. †SEX: Male ☐ Female ☐ 5. AGE ____/____/____ Years Months Days 6. DATE OF DEATH ____/____/____ Day Month Year

7. MARITAL STATUS: (*a*) Married ☐ (*b*) Divorced ☐ (*c*) Single ☐ (*d*) Widowed ☐

8. PLACE OF DEATH ____________________ / ____________
Sub-location or estate and town Sub-county

9. USUAL RESIDENCE ____________________ / ____________
Sub-location or estate and town Sub-county

10. LEVEL OF EDUCATION ____________ 11. OCCUPATION ____________

12A. NATURAL CAUSES*

Malaria	☐	Anaemia	☐	Cancer	☐
Pneumonia	☐	Jaundice	☐	Urinary Obstruction	☐
Measles	☐	Child/pregnancy/birth	☐	AIDS	☐
Tetanus	☐	Sudden death	☐	Malnutrition	☐
Tuberculosis	☐	Alcoholism	☐	Asthma	☐

Other known cause, specify ____________________

I am satisfied after the above-mentioned death is not one to which section 386 or 387 of the Criminal Procedure Code (Cap.75) apply. An external examination of the body has/has not been made by a medical practitioner.

12B. UNNATURAL CAUSES*

Accident	☐	Motor Vehicle	☐	House fire	☐
Poisoning	☐	Attacked by animal or snake	☐		
Suicide	☐	Drowning	☐	Other known cause, specify ______	

I certify that provisions of *Cap.* 75 have been observed.
Name ____________ Date ____________ Signature ____________
(*Police Officer or Magistrate*)

13. NAME ____________________
First Name ______ Middle Name ______ *Father's or husband's name ______

14. CAPACITY OF INFORMANT
RELATIVE ☐ VILLAGE ELDER ☐ Other, specify ____________

15. DATE ____________ 16. SIGNATURE OF INFORMANT ____________

17. DATE ____________ 18. REGISTRATION ASSISTANT FOR: ____________ 19. SIGNATURE ____________
Day/Month/Year (*Name of Sub-location*)

20. SUB-COUNTY ____________ 21. REGISTRATION No. ____________

22. DATE ____________ 23. NAME ____________ 24. SIGNATURE ____________

*If the deceased was a married woman, husband's name can be written, †cross the appropriate box, thus x

GPK (SP) 7105—80m Bks.—8/14
