# Supplementary material for: Mortuary and hospital-based HIV mortality surveillance among decedents in a low-resource setting: lessons from Western Kenya
Source: BMC Public Health. 2022 Mar 29;22:609. doi: 10.1186/s12889-022-12909-3 (PMC8962591; doi:10.1186/s12889-022-12909-3)
Supplement: Supplementary file 9 — Additional file 9. Sample Manifest for Blood Specimen. Used to track sample transport to the laboratory. [file 12889_2022_12909_MOESM9_ESM.pdf]

# Mortality Surveillance Study

## Sample Manifest for Blood Specimen

**Facility name**: ________________________________________________ **Date**:___________________________

| S.No | Blood Specimen ID | Date Collected (DD/MM/YY) | Time Received at CRC lab (HH/MM) | Samples Labelled Well (Yes/No) | Samples Correctly packaged Yes/No | Quality Code (s) | CRC lab staff receiving or Rejecting samples (initials) | Comments |
|---|---|---|---|---|---|---|---|---|
| | | | | | | | | |
| | | | | | | | | |
| | | | | | | | | |
| | | | | | | | | |
| | | | | | | | | |

**Quality Codes:**

1) Acceptable – No Rejection
2) Broken/cracked/open container
3) Haemolysis
4) Leaking container
5) Insufficient blood
6) Clotting
7) Incorrectly labelled/unlabelled
8) Specimen lost
9) Specimen mix-up
10) Others (specify)

v 16.08.18
