# Supplementary material for: Mortuary and hospital-based HIV mortality surveillance among decedents in a low-resource setting: lessons from Western Kenya
Source: BMC Public Health. 2022 Mar 29;22:609. doi: 10.1186/s12889-022-12909-3 (PMC8962591; doi:10.1186/s12889-022-12909-3)
Supplement: Supplementary file 10 — Additional file 10. COD panelist summary form. Used to record immediate, antecedent and underlying causes of death. [file 12889_2022_12909_MOESM10_ESM.pdf]

v 11.Aug2019

COD panel form

Surveillance of HIV-associated mortality in Kenya: Mortuary and hospital-based surveillance in Kisumu County

Review O First Review O For QC O Panel

Part A: Case summary
Study ID [ ][ ][ ][ ][ ] *from mortuary register*
Case details:
Sex: O Male O Female

Age at death: ________
Date of death: ____________
Date of most recent hospitalization before death: ____________
**days hospitalized auto calculated*
If date of hospitalization is missing, approximate days/months hospitalized: ________

Summary of conditions, medical history and diagnosis before death:
Summarize the case as if writing a discharge or death note for a patient's chart.

Hospitalization before death
Approximate time interval from onset of symptoms during hospitalization leading to death ________
Time units: O Hours O Days O Weeks O Months O Years

Patient's major complaints to attending medic during most recent hospitalization
___

Direct or immediate cause of death (If recorded in clinical notes) ____________________

____
Due to

____

Due to

____

Due to

____

Other noted diagnosis

____

Notes on sequence

Part B: Cause of death determination

Mortuary:

☐ **1**- JOOTRH

2 - Kisumu County Referral Hospital

Study ID [ ][ ][ ][ ][ ] *from mortuary register*

Case details:

Demographics

Specify; Years [Y], Months [M] , Weeks [W], OR Days [D]

Sex: O Male O Female O Unknown

Age: [ ] [ ]

Deceased DOB: [ ][ ]/[ ][ ]/[ ][ ][ ][ ]

Date of death: [ ][ ]/[ ][ ]/[ ][ ][ ][ ]
DD/MM/YYYY

HIV Status Positive ☐
Negative ☐

Date of most recent hospitalization before death: ____________

**days hospitalized auto calculated*

If date of hospitalization is missing, approximate days/months hospitalized: ________

Panelist COD determination

Disease or condition directly leading to death

IMMEDIATE CAUSE: disease or condition directly leading to death (a)

[ ] ICD-10 CODE [ ][ ][ ]-[ ]

Due to:

ANTECEDENT CAUSE: Morbid conditions, if any, which gave rise to **immediate cause** (b)

[ ] ICD-10 CODE [ ][ ][ ] [ ]

ANTECEDENT CAUSE: Due to, stating the underlying cause last (c)

[ ] ICD-10 CODE [ ][ ][ ] [ ]

OTHER SIGNIFICANT CONDITIONS: Contributing to death but not related to (a)

[ ]

Conclusion

Was cause of death determined? O Yes O No (if no, indicate why) ________________________

Why panelist was unable to determine cause of death: O Insufficient data O Inconclusive data

Public health action recommendations

Could this death have been prevented? O Yes O No (if no, indicate why) ________________

Recommendations for public health:____

Panelist consensus results (for QC cases needing panel discussions)

O Unanimous O Non-consensus (*Unanimous = 100%; Non-consensus=50% or less*)

List contributing factors/reasons why consensus was not reached for immediate COD (e.g. not enough data, different interpretation of results) ____

Part C: HIV status documentation and association with mortality

HIV status documentation

a	Known positive at hospitalization	Yes O No O	(if yes, go to d: if No, go to b)
b	If no, were they tested for HIV?	Yes O No O	
c	If tested, HIV test result	Positive O Negative O Unknown O	
d	On HAART?	Yes O No O	

HIV symptoms documentation in patient chart

Conditions where a presumptive diagnosis can be made on the basis of clinical signs or simple investigations

QN	Sign/symptom/illness	Documented?
1	HIV wasting syndrome	Yes **O** No **O**
2	Pneumocystis pneumonia	Yes **O** No **O**
3	Recurrent severe or radiological bacterial pneumonia	Yes **O** No **O**
4	Chronic herpes simplex infection (orolabial, genital or anorectal of more than one month's duration)	Yes **O** No **O**
5	Oesophageal candidiasis	Yes **O** No **O**
6	Extrapulmonary TB	Yes **O** No **O**
7	Kaposi's sarcoma	Yes **O** No **O**
8	Central nervous system (CNS) toxoplasmosis	Yes **O** No **O**
9	HIV encephalopathy	Yes **O** No **O**
10	Extrapulmonary cryptococcosis including meningitis	Yes **O** No **O**
11	Disseminated non-tuberculous mycobacteria infection	Yes **O** No **O**
12	Progressive multifocal leukoencephalopathy (PML)	Yes **O** No **O**
13	Candida of trachea, bronchi or lungs	Yes **O** No **O**
14	Cryptosporidiosis	Yes **O** No **O**
15	Isosporiasis	Yes **O** No **O**
16	Visceral herpes simplex infection	Yes **O** No **O**
17	Cytomegalovirus (CMV) infection (retinitis or of an organ other than liver, spleen or lymph nodes)	Yes **O** No **O**
18	Any disseminated mycosis (e.g. histoplasmosis, coccidiomycosis, penicilliosis)	Yes **O** No **O**
19	Recurrent non-typhoidal salmonella septicaemia	Yes **O** No **O**
20	Lymphoma (cerebral or B cell non-Hodgkin)	Yes **O** No **O**
21	Invasive cervical carcinoma	Yes **O** No **O**
22	Visceral leishmaniasis	Yes **O** No **O**

Was the death HIV associated? Yes O No O
